# κ-Opioid Signaling in the Lateral Hypothalamic Area Modulates Nicotine-Induced Negative Energy Balance

**DOI:** 10.3390/ijms22041515

**Published:** 2021-02-03

**Authors:** Patricia Seoane-Collazo, Amparo Romero-Picó, Eva Rial-Pensado, Laura Liñares-Pose, Ánxela Estévez-Salguero, Johan Fernø, Rubén Nogueiras, Carlos Diéguez, Miguel López

**Affiliations:** 1 Department of Physiology, CiMUS, University of Santiago de Compostela-Instituto de Investigación Sanitaria, 15782 Santiago de Compostela, Spain; amparo.romero@usc.es (A.R.-P.); eva.pensado@usc.es (E.R.-P.); laura.linares@usc.es (L.L.-P.); anxela.estevez@usc.es (Á.E.-S.); ruben.nogueiras@usc.es (R.N.); carlos.dieguez@usc.es (C.D.); 2 CIBER Fisiopatología de la Obesidad y Nutrición (CIBERobn), 15706 Santiago de Compostela, Spain; 3 Hormone Laboratory, Haukeland University Hospital, N-5021 Bergen, Norway; johan.ferno@mbi.uib.no

**Keywords:** nicotine, kappa opioid receptor, lateral hypothalamus, AMPK, mTOR

## Abstract

Several studies have reported that nicotine, the main bioactive component of tobacco, exerts a marked negative energy balance. Apart from its anorectic action, nicotine also modulates energy expenditure, by regulating brown adipose tissue (BAT) thermogenesis and white adipose tissue (WAT) browning. These effects are mainly controlled at the central level by modulation of hypothalamic neuropeptide systems and energy sensors, such as AMP-activated protein kinase (AMPK). In this study, we aimed to investigate the kappa opioid receptor (κOR)/dynorphin signaling in the modulation of nicotine’s effects on energy balance. We found that body weight loss after nicotine treatment is associated with a down-regulation of the κOR endogenous ligand dynorphin precursor and with a marked reduction in κOR signaling and the p70 S6 kinase/ribosomal protein S6 (S6K/rpS6) pathway in the lateral hypothalamic area (LHA). The inhibition of these pathways by nicotine was completely blunted in κOR deficient mice, after central pharmacological blockade of κOR, and in rodents where κOR was genetically knocked down specifically in the LHA. Moreover, κOR-mediated nicotine effects on body weight do not depend on orexin. These data unravel a new central regulatory pathway modulating nicotine’s effects on energy balance.

## 1. Introduction

Smoking has, for a long time, been associated with a leaner phenotype. Nicotine, the main bioactive compound of tobacco, is behind this effect due to its ability to regulate both food intake and energy expenditure [1]. Nicotine exerts its anorectic effect by the modulation of the hypothalamic neuropeptide systems [2,3], and increases the energy expenditure by promoting the activation of brown adipose tissue (BAT) thermogenesis and the browning of white adipose tissue (WAT) [3,4,5,6]. So far, the energy sensor AMP-activated protein kinase (AMPK) at the central level has emerged as a mediator of several peripheral signals and compounds associated with the regulation of energy homeostasis [7,8,9,10,11,12,13], including nicotine [3,6,14]. Although nicotine’s effects are mediated by nicotinic acetylcholine receptors (nAChR), mainly α3β4 nAChRs [2,3,15], we found that the kappa opioid receptor (κOR) is also necessary for the nicotine-induced activation of BAT thermogenesis and browning of WAT [6]. Nevertheless, the mechanistic aspects of this interaction remain unexplored.

κOR is specifically activated by endogenous opioids derived from prodynorphin [16]. κOR in the central nervous system has already been reported as a regulator of energy intake and body weight [17,18]. However, little is known about the intracellular signaling pathways mediating the metabolic effects of the dynorphin/κOR system. κOR signaling pathways, including the members of the mitogen-activated protein kinase (MAPK) family, p38 and p44/42 MAPK (Erk1/2), have been shown to modulate the phosphorylation of p70 S6 Kinase (S6K) [19,20]. This mitogen-activated Ser/Thr protein kinase is primarily required for cell growth and the regulation of protein synthesis, and has been reported to play a relevant role in the control of energy balance [21,22,23]. In this sense, κOR system activation and the crosstalk between ERK and the S6K/ribosomal protein S6 (rpS6) pathway in LHA modulate the acute orexigenic action of ghrelin [18] and melanin-concentrating hormone (MCH) [24]. The relevance of κOR as a drug target was again put forward following data showing that pharmacological inhibition of κOR, but not of the ẟ- (δOR) or the μ opioid (µOR) receptor subtypes, fully blocked calorie restriction-induced hypothermia and increased weight loss [25]. Additional data have also involved the S6K/rpS6 pathway in the modulation of BAT thermogenesis [23].

In the present study, we used a combination of pharmacological and genetic approaches to provide evidence that nicotine treatment increases energy expenditure through the inhibition of opioid signaling and the S6K/rpS6 pathway in the lateral hypothalamic area (LHA). These results endorse κOR signaling in the LHA as a key modulator of nicotine’s catabolic action.

## 2. Results

### 2.1. κOR Mediates Nicotine-Induced Regulation of Hypothalamic AMPK

**Figure 1:**
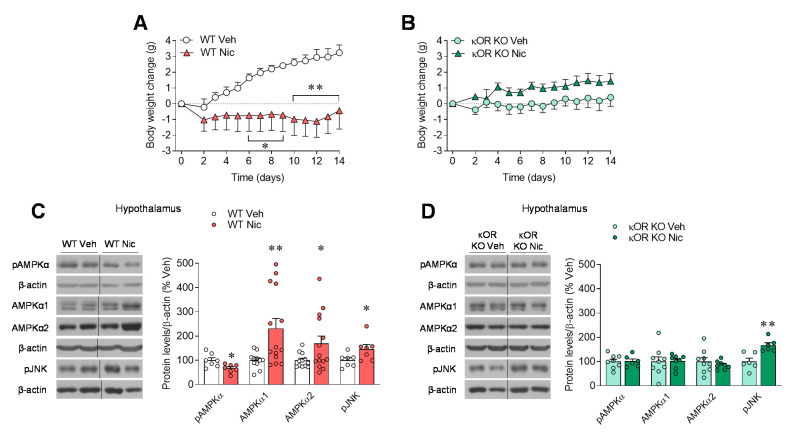
Nicotine’s effect on energy balance and hypothalamic AMPK in κOR null mice. (**A**,**B**) Body weight change (*n* = 7 mice/group), (**C**,**D**) protein levels of AMPK and pJNK in the hypothalamus (*n* = 6–9 mice/group), of WT (**A**–**C**) or κOR KO (**B**,**C**) mice treated subcutaneously (SC) with vehicle or nicotine through osmotic minipumps for 14 days. * *p* < 0.05, ** *p* < 0.01 vs. vehicle. Data are expressed as mean ± SEM. The bands in gels from panels **C** and **D** have been spliced from the same original gels.

We previously demonstrated that κOR is essential for the ability of nicotine to exert a negative energy balance, specifically by inducing BAT thermogenesis and browning of WAT [6]. We confirmed those results in the current study, because chronic administration of nicotine reduced weight gain in WT mice but not in mice with global genetic ablation of κOR (Figure 1A,B). Similar results were observed after chronic pharmacological inhibition of κOR by daily intracerebroventricular (ICV) injection of the κOR antagonist norBNI for 14 days (Supplementary Materials Figure S1A–D). Next, we focused on the central mechanism that could mediate nicotine’s effect on body weight through κOR. In previous studies, we found that hypothalamic AMPKα is a key regulator of nicotine’s negative energy balance in rats [3,6]. Indeed, WT mice treated with nicotine reduced hypothalamic phosphorylated AMPKα (pAMPKα) protein levels accompanied by an increase in non-phosphorylated AMPKα isoforms when compared with vehicle-treated mice (Figure 1C). This effect in the hypothalamic AMPK pathway was blunted in κOR mutant mice (Figure 1D). In addition, in both genotypes we observed that nicotine treatment increased phospho-stress-activated protein kinase/c-Jun N-terminal kinase (SAPK/JNK) (pJNK) (Figure 1C,D), a member of the MAPK family that has been extensively linked to the development of obesity, type-2 diabetes, and related comorbidities [13,26,27].

### 2.2. The Effect of Nicotine on Body Weight Is Independent of OX Signaling 

**Figure 2:**
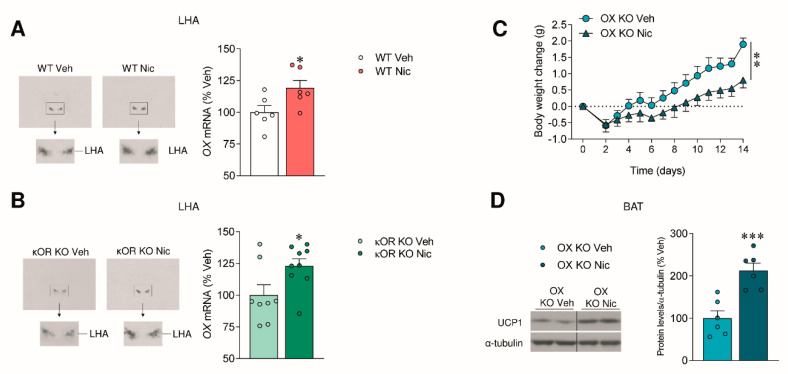
Nicotine’s effect on orexin in the lateral hypothalamic area (LHA). (**A**,**B**) OX mRNA levels in the LHA (*n* = 6–9 mice/group) in WT (**A**), and κOR KO (**B**), mice treated SC with vehicle or nicotine through osmotic minipumps for 14 days. (**C**) Body weight change (*n* = 6 mice/group), (**D**) protein levels of UCP1 in the BAT (*n* = 6 mice/group) of OX KO mice treated SC with vehicle or nicotine through osmotic minipumps for 14 days. * *p* < 0.05, ** *p* < 0.01 vs. *** *p* < 0.001 vehicle. Data are expressed as mean ± SEM. The bands in gels from panel **D** have been spliced from the same original gels.

Findings from our group have linked the AMPK pathway in the ventromedial hypothalamic nucleus (VMH) and OX in LHA via orexin receptor 1 (OX1R), as a new physiological mechanism that controls BAT thermogenesis and energy balance [12]. In addition, evidence suggests that OX1R activation modulates the κOR function [28], and dynorphin neurons and OX neurons are highly co-localized in the LHA [29]. To gain further insight into the mechanism mediating nicotine effects through κOR, we next analyzed OX levels in the hypothalamus of WT and κOR KO mice. We found that nicotine increased OX levels independently of κOR (Figure 2A,B). Next, we treated OX KO mice with nicotine. OX deletion did not alter nicotine’s effects on body weight (Figure 2C) and uncoupling protein 1 (UCP1) levels in BAT (Figure 2D). These results suggest that, despite its increased levels in nicotine-treated animals, OX is not relevant for mediating the catabolic effect of nicotine, and κOR signaling is independent of orexin. 

### 2.3. Nicotine Inhibits Prodynorphin

**Figure 3:**
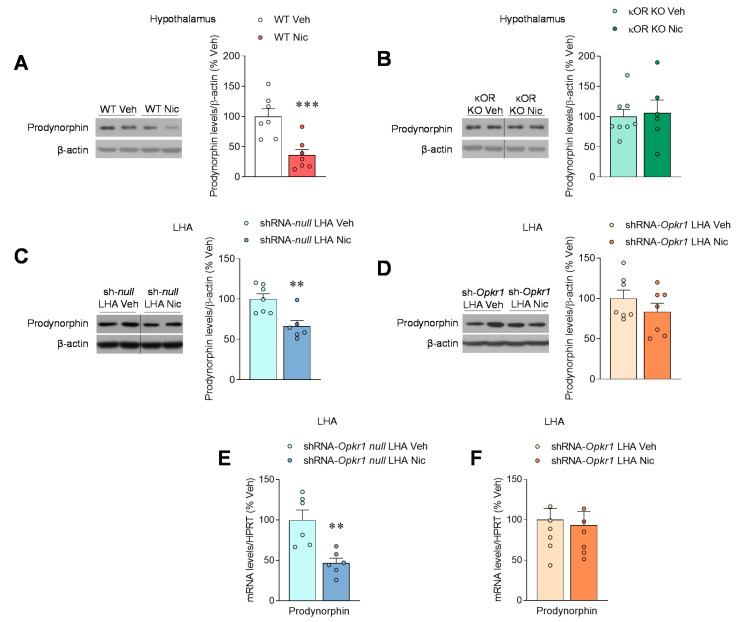
Nicotine effects on prodynorphin in the LHA. (**A**,**B**) Protein levels of prodynorphin in the hypothalamus (*n* = 6–8 mice/group) of WT (**A**), or κOR KO (**B**), mice treated SC with vehicle or nicotine through osmotic minipumps for 14 days. (**C**,**D**) Protein levels of Prodynorphin in the LHA (*n* = 6–7 rats/group), (**E**,**F**) mRNA levels of prodynorphin in the LHA (*n* = 6–8 rats/group), of rats stereotaxically treated within the LHA with shRNA-*null* (**C**,**E**) or shRNA-*Opkr1* (**D**,**F**) adeno-associated viruses and treated SC with vehicle or nicotine through osmotic minipumps for 14 days. ** *p* < 0.01 vs. *** *p* < 0.001 vehicle. Data are expressed as mean ± SEM. The bands in gels from panels **B** and **C** have been spliced from the same original gels.

As previously reported for other drugs of abuse, such as cocaine [30,31], nicotine reduces protein levels of the endogenous κOR ligand precursor prodynorphin in the hypothalamus (Figure 3A) when compared with vehicle-treated mice, while this effect was blunted in κOR mutant mice (Figure 3B). 

To gain further insight in the actions of κOR in the LHA, we proceeded to the selective knockdown of this receptor using stereotaxic administration of AAV expressing a shRNA against *Opkr1* (shRNA-*Opkr1*), the gene encoding κOR [6,18]. The efficiency of the knockdown was validated by the decreased *Opkr1* mRNA expression and/or κOR protein content in the LHA (shRNA-*null*: 100 ± 8.54; shRNA-*Opkr1*: 68.5 ± 3.4; *p* < 0.01). In keeping with the data obtained in κOR null mice (Figure 1A,B) and norBNI (Figure S1A), genetic targeting of κOR in the LHA blunted the weight-reducing effect of nicotine (Figure S2A,B). Our data showed that both protein and mRNA prodynorphin levels were diminished in the LHA after nicotine treatment in control animals (Figure 3C,E). However, these changes were not detected in rats treated with nicotine after *Opkr1* silencing (Figure 3D,F). Furthermore, we aimed to investigate whether AMPK in the LHA might be involved in the effects of nicotine. Our data showed that pAMPKα levels were not modified by nicotine treatment either in sh-*null* or sh-*Opkr1* treated rats (Figure S2C–D), suggesting that AMPKα in the LHA is not involved in the mediation of nicotine effects. Overall, these data indicate that nicotine exerts highly nuclei-specific effects within the hypothalamus. 

### 2.4. Inhibition of Opioid Signaling Mediates Nicotine Negative Energy Balance

**Figure 4:**
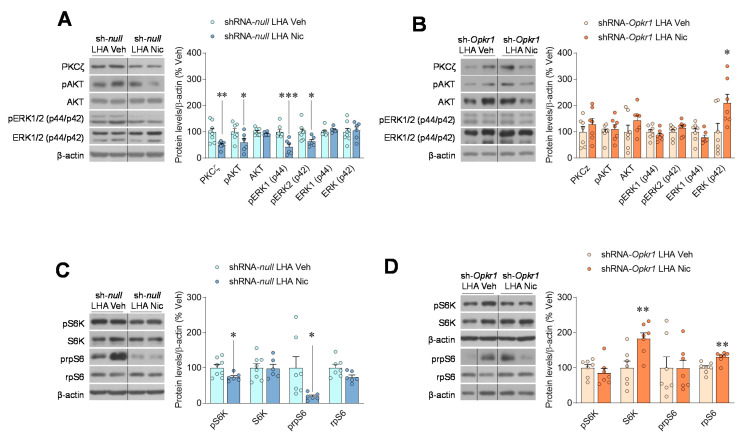
Nicotine inhibits κOR signaling in the LHA. (**A**,**B**) Protein levels of κOR signaling pathways in the LHA (*n* = 6–8 rats/group), (**C**,**D**) protein levels of S6K signaling pathway in the LHA (n= 6–8 rats/group), of rats stereotaxically treated within the LHA with shRNA-*null* (**A**–**C**) or shRNA-*Opkr1* (**B**–**D**) adeno-associated viruses and treated SC with vehicle or nicotine through osmotic minipumps for 14 days. * *p* < 0.05, ** *p* < 0.01 vs. *** *p* < 0.001 vehicle. Data are expressed as mean ± SEM. The bands in gels from panels **A**–**D** have been spliced from the same original gels.

Finally, we analyzed key downstream targets of κOR opioid signaling pathway. We observed an inhibition of opioid signaling in the intact LHA of nicotine-treated rats, as demonstrated by decreased protein kinase C zeta (PKCζ), phosphorylated protein kinase B (pAKT), and pERK1/2 (Figure 4A). However, nicotine failed to exert all these effects when κOR was knocked down in the LHA (Figure 4B). PKCζ and AKT have been found to increase the phosphorylation of pS6K1 [32]. Accordingly, the phosphorylated levels of S6K and its downstream target rpS6, (which has been linked to BAT activation [23]) were significantly decreased after the treatment with nicotine (Figure 4C), an effect that was blunted when *Opkr1* was knocked down specifically in the LHA (Figure 4D). 

## 3. Discussion

**Figure 5:**
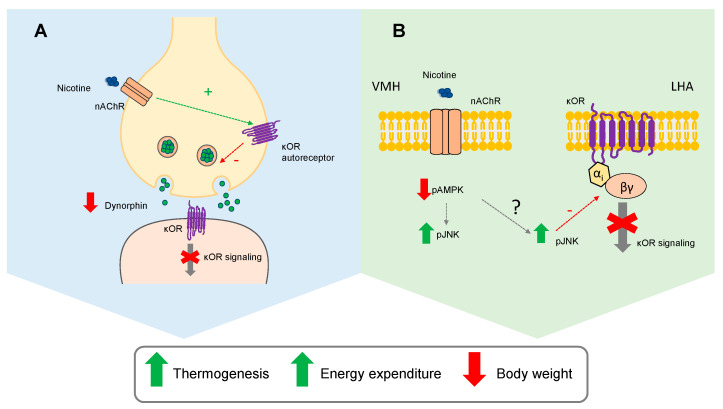
κOR in the LHA as a mediator of nicotine actions on energy balance. Nicotine-induced activation of thermogenesis and the consequent increase in energy expenditure and decreased body weight could be explained by two models involving κOR. (**A**) Nicotine signaling by an unknown mechanism would activate κOR auto-receptors at the presynaptic level, leading to an inhibitory feedback of the release of dynorphin peptides, thus reducing postsynaptical κOR signaling. (**B**) Nicotine activates hypothalamic JNK, which, by a mechanism involving structural changes in the κOR signaling complex, would inhibit κOR downstream effectors. The mechanism by which nicotine increases JNK in the LHA is unknown but could be mediated by changes in AMPK, in response to nicotine treatment. AMPK, AMP-activated protein kinase; JNK, c-Jun N-terminal kinase; κOR, Kappa opioid receptor; LHA, lateral hypothalamic area; nAChR, nicotinic acetylcholine receptor; VMH, ventromedial hypothalamic nucleus.

In this study, we showed that nicotine-induced weight loss and associated UCP1 increase in BAT rely on an intact κOR signaling in the LHA. This is because disruption of κOR by genetic (global genetic depletion or virogenetic knockdown of κOR in the LHA) and pharmacological (ICV injection of the κOR antagonist norBNI) tools were all effective in blocking nicotine’s actions on energy homeostasis. Moreover, we have also elucidated the mechanism by which κOR modulates nicotine’s effects on energy expenditure: (i) nicotine inhibits dynorphin and κOR signaling in the LHA; and (ii) an intact κOR signaling is necessary for nicotine’s catabolic effect (Figure 5).

Nicotine triggers sympathetic nerve system tone to BAT through the modulation of pAMPKα in the VMH [3,6]. In agreement with previous results, we observed that pAMPKα levels were reduced in the hypothalamus of WT, but not in κOR KO mice. One important thing to take into consideration is that the different hypothalamic nuclei execute discrete functions, and the mediators of these functions, as observed in the case of κOR and AMPK, are therefore region-specific [6,24,33]. Thus, we analyzed the effect of nicotine on the AMPK pathway in a nucleus-specific way. The reduction in pAMPKα by nicotine that occurs within the VMH [3,6] does not extend to the LHA, as demonstrated by our current data. This is of relevance because it indicates that nicotine is acting through different molecular mechanisms in different nuclei to modulate energy balance. Further work will be needed to address whether those molecular pathways are connected or represent independent mechanisms. 

We have previously reported a functional link between AMPK in the VMH and the LHA (through a glutamatergic-dependent mechanism and OX neurons), mediating bone morphogenic protein 8B (BMP8B) effects in energy homeostasis [9,12]. Given that: (i) OX signaling regulates several aspects of BAT, such as differentiation and activation [34,35]; and (ii) OX is highly colocalized with dynorphin in the LHA [29], we hypothesized that OX might play a role in the modulation of nicotine effects thought opioid signaling. However, increased levels of OX in the LHA were observed after nicotine treatment both in WT and in κOR mice, demonstrating that the effect is independent of this opioid receptor. In addition, nicotine treatment in OX KO mice still promoted a negative energy balance, indicating that nicotine-induced increase in OX may not be crucial for nicotine’s actions on body mass. 

Next, we decided to investigate if the expression of dynorphin, the natural ligand of κOR, was modulated by nicotine. We observed a clear inhibition of prodynorphin expression by nicotine treatment in the presence of a functional κOR. Interestingly, in κOR KO mice or rats where *Opkr1* was specifically downregulated in the LHA, prodynorphin levels remained unaltered after nicotine treatment. Given the existence of a presynaptic inhibitory feedback regulating the release of opioids peptides, including dynorphin [36,37], these results suggest that nicotine may activate presynaptic κOR auto-receptors, leading to the inhibition of dynorphin and consequent inhibition of postsynaptic κOR signaling (Figure 5). Another mechanism by which nicotine could be decreasing κOR signaling may be partially shared with long-duration κOR antagonists, such as norBNI, that have been found to disrupt κOR signaling by increased phosphorylation of JNK [38]. JNK activation inhibits the κOR, µOR, and dopamine 2 receptors by recruitment of peroxiredoxin 6 to the receptor-Gαi complex, promoting a long-lasting structural change in the receptor signaling complex [39]. It was also proposed that OX1R inhibition of κOR in a JNK-dependent fashion may be explained by this mechanism [28,39]. In our study, nicotine-induced activation of hypothalamic JNK was observed in both WT and κOR KO mice, but inhibition of opioid signaling was observed only when κOR was intact. We hypothesized that the mechanism by which pJNK is elevated after nicotine treatment could be dependent on AMPK (Figure 5). In this sense, inhibition or activation of AMPK in the VMH has been proved to increase or decrease JNK activation, respectively [13]. Given that expression of a constitutively active form of AMPKα in the VMH abolished nicotine-induced negative energy balance [3] further studies to address the interaction between VMH-AMPK and JNK expression in the LHA are merited.

Inhibition of κOR systems by nicotine to induce a negative energy balance agrees with previous reports of κOR KO mice showing elevated energy expenditure and higher locomotor activity, associated with reduced body weight and adiposity [17]. In our study, we observed downregulation of PKCζ and subsequent decrease in the activation of ERK1/2, as previously described [40,41,42], in nicotine-treated rats but not in rats with defective κOR expression. In addition, we observed a clear inhibition of the S6K1/rpS6 pathway in the LHA, which has been reported as a central mechanism modulating BAT thermogenesis [23]. Our results do not contradict previous reports showing an opposite regulation of pERK and S6K [20]. This discrepancy could be explained by differences in acute vs. chronic modulation of these pathways; S6K was found to be regulated by ERK signaling in an early phase of the acute control of food intake, with the molecular changes in these pathways already being restored before the peak orexigenic response [24]. In addition, S6K can be phosphorylated by downstream kinases to phosphatidylinositol 3-kinase, by a mediator of opioid signaling cascades, including PKCζ and AKT [32], that are downregulated in the LHA after nicotine treatment when κOR is intact. In this sense, a possible improvement of the current study would be a better dissection of the κOR downstream pathways mediating the observed actions of nicotine. Further studies based on the genetic knockdown of specific proteins, such as JNK1, in specific neuronal cell types and non-neuronal cells, complemented by single cell analysis and detailed metabolic phenotyping are now needed to clarify the involvement of this opioid receptor on nicotine actions. 

In summary, we describe that the catabolic actions of nicotine depend, at least in part, on the prodynorphin/κOR system. Besides its action on AMPK in the VMH, nicotine disrupts κOR signaling in the LHA by inhibiting the precursor of its endogenous ligand dynorphin. Our study shows that inhibition of κOR signaling by nicotine treatment in the LHA induces a negative energy balance, associated to repressed S6K/rpS6 signaling. Overall, this evidence reveals what appears to be a key component of the intricate pathways affected by nicotine to induce a negative energy balance. Dissecting out the signaling pathway involved may provide a more specific drug targeting for obesity.

## 4. Materials and Methods

### 4.1. Animals

Adult male Sprague-Dawley rats (8–10-weeks-old, 200–250 g; Animalario General USC, Santiago de Compostela, Spain), C57BL6 adult male mice (8–12-weeks-old, 20–25 g Animalario General, USC, Santiago de Compostela, Spain), adult male null κ opioid receptor (κOR KO) mice, adult male null orexin (OX KO) mice and their respective wild-type controls (8–12-weeks-old, 20–25 g) were used. The animals were housed under constant conditions of humidity and temperature (22–24 °C) with a 12-h light–dark cycle (08:00 to 20:00) and allowed free access to water and a standard laboratory diet (SAFE A04: 3.1% fat, 59.9% carbohydrates, 16.1% proteins, 2.791 kcal g^−1^; *Scientific Animal Food and Engineering*; Nantes, France). Adeno-associated virus (AAV) administration, osmotic minipumps, and cannulae implantation were performed under intraperitoneal ketamine/ xylazine anesthetics (ketamine 80 or 8 and xylazine 100 or 3 mg/kg body weight for rats or mice, respectively). The animals were euthanatized, and all the tissues were removed rapidly, frozen immediately on dry ice, and kept at −80 °C until analysis or fixed in formalin 10% and lately paraffin-embedded. All experiments were performed according to the International Law on Animal Experimentation and were approved by the USC Ethical Committee.

### 4.2. Subcutaneous Treatment

κOR KO [6,18,24], OX KO [12] mice and their respective WT were housed individually and implanted subcutaneously (SC) with an osmotic minipump flow moderator (Model 1002, Alzet, DURECT Corporation; Cupertino, CA, USA) for the infusion of a daily dose of 25 mg/kg of nicotine (nicotine–hydrogen–tartrate salt, Sigma-Aldrich; St Louis, MO, USA) or saline. The osmotic minipumps were inserted in a subcutaneous pocket on the dorsal surface created using blunt dissection and closed with surgical sutures. Body weight was measured daily for 14 days. 

### 4.3. Intracerebroventricular Treatment 

Mice were housed individually and implanted subcutaneously with an osmotic minipump flow moderator (Model 1002, Alzet, DURECT Corporation; Cupertino, CA, USA) containing 25 mg/kg/day of nicotine or saline. ICV treatment of κOR antagonist norBNI (nor-Binaltorphimine dihydrochloride; 4.16 µg/µL dissolved in saline; Tocris Bioscience; Bristol, UK) or saline was administered through an osmotic minipump flow moderator (Model 1002, Alzet, DURECT Corporation; Cupertino, CA, USA) connected through a catheter tube to brain infusion cannulae (Brain Infusion Kit 3, Alzet, DURECT Corporation; Cupertino, CA, USA). Food and body weight were measured daily for 14 days and correct positioning in the lateral ventricle was confirmed by postmortem histological examination. 

### 4.4. Stereotaxic Microinjection of Viral Vectors

Rats were placed in a stereotaxic frame (David Kopf Instruments; Tujunga, CA, USA) and the LHA was targeted bilaterally using a 25-gauge needle (Hamilton; Reno, NV, USA) using the following stereotaxic coordinates: 2.85 mm posterior to the bregma, ±2 mm lateral to the midline and 8.1 mm ventral [6]. One µl at each injection site of AAV vectors (1 × 10^9^ genomic copies µL^−1^) encoding *null* or *Opkr1* short-hairpin RNAs [6,18] were delivered at a rate of 200 nL min^−1^ for 5 min. Fourteen days after AAV injection, an osmotic minipump flow moderator (Model 2002, Alzet, DURECT Corporation; Cupertino, CA, USA) containing a daily dose of 4 mg/ kg of nicotine (nicotine–hydrogen–tartrate salt, Sigma-Aldrich; St Louis, MO, USA) or saline was implanted subcutaneously. Food and body weight were measured for 14 days.

### 4.5. Real-Time Quantitative RT-PCR

Real-time PCR (TaqMan^®^; Applied Biosystems; Foster City, CA, USA) was performed using specific primers and probes for uncoupling protein 1 (*Ucp1*; NM_009463, Fw-primer 5′-CAATGACCATGTACACCAAGGAA-3′; Rv-primer 5′-GACCCGAGTCGCAGAAAAGAA-3′; Probe FAM-5′-ACCGGCAGCCTTTTTCAAAGGGTTTG3’-TAMRA) [6,13]. Trizol Reagent (Invitrogen; Carlsbad, CA, USA) was used for the isolation of total RNA according to the supplier’s protocol. cDNA synthesis was performed with M-MLV enzyme (Invitrogen; Carlsbad, CA, USA) following the manufacturer’s protocol. Values were expressed in relation to 18S ribosomal RNA levels (*Rn18s*; NR_003278.3, Fw-primer 5′-CGGCTACCACATCCAAGGAA-3′; Rv-primer 5’-GCTGGAATTACCGCGGCT-3′; Probe FAM-5′-GACGGCAAGTCTGGTGCCAGCA3’-TAMRA) or HPRT (*Hprt* NM_012583, Fw-Primer 5′-AGCCGACCGGTTCTGTCAT-3′; Rv-Primer 5′-GGTCATAACCTGGTTCATCATCAC-3′; Probe FAM-5′-CGACCCTCAGTCCCAGCGTCGTGAT3′-TAMRA).

### 4.6. Hematoxylin-Eosin Staining and Immunohistochemistry

Gonadal WAT samples were fixed for 24 h in 10% formalin buffer and then dehydrated and embedded in paraffin by a standard procedure. Sections of 3 µm were made in a microtome and stained using a standard hematoxylin/eosin (BioOptica; Milano, Italy) following a procedure according to the manufacturers’ instructions, as previously described [6]. Immunohistochemical analysis of UCP1 was performed using an anti-UCP1 antibody (1:500; ab10983; Abcam; Cambridge, UK) and the detection was performed with an anti-rabbit antibody conjugated with Alexa 488 (1:200; Molecular Probes; Grand Island, NY, USA) as previously described [6]. Images were obtained using a digital camera Olympus XC50 (Olympus Corporation; Tokyo, Japan) at 40× magnification for mice. Adipocyte area was measured on hematoxylin and eosin-stained slides using Adiposoft 1.16 (CIMA; University of Navarra, Pamplona, Spain), and UCP1 stained area in immunohistochemical slides was measured using ImageJ-1.53 software (National Institutes of Health, Bethesda, MD, USA). 

### 4.7. Western Blotting

Protein lysates were subjected to SDS-PAGE, electrotransferred to polyvinylidene difluoride membranes (PVDF; Merck Millipore; Billerica, MA, USA) and probed successively with the following antibodies: UCP1 (1:10,000; ab10983); PKCζ (1:1000; ab59364) (Abcam; Cambridge, UK); β-actin (1:5000; A5316), α-tubulin (1:5000; T5168), (Sigma-Aldrich, St. Louis, MO, USA); AMPKα1 (1:1000; 07–350), AMPKα2 (1:1000; 07–363) (Millipore; Billerica, MA, USA); phospho-AKT (Ser473) (1:1000; 9271), AKT (1:1000; 9272), phospho-AMPKα (Thr172) (1:1000; 2535S), phospho-p44/42 MAPK (ERK1/2) (Thr202/Tyr204) (pERK 1-2) (1:1000; 4370), phospho-JNK (T183/Y185) (1:1000; 4671S), phospho-S6K (Thr389) (1:1000; 9205), S6K (1:1000; 9202), phospho-rpS6 (Ser235/236) (1:1000; 2211), rpS6 (1:1000; 2217) (Cell Signaling; Danvers; MA, USA) after incubating the membranes with 3% BSA blocking buffer. Specific antigen–antibody bindings were detected using horseradish-peroxidase-conjugated secondary antibodies (Dako Denmark; Glostrup, Denmark) and an enhanced chemiluminescence detection method according to the manufacturer’s instructions (Pierce ECL Western Blotting Substrate; Thermo Scientific, Waltham, MA, USA) as previously described [13,43]. Autoradiographic films (Fujifilm; Tokyo, Japan) were scanned and the band’s signal was quantified by densitometry using ImageJ-1.53 software (National Institutes of Health, Bethesda, MD, USA). Values were expressed relative to β-actin (hypothalamus, LHA) or α-tubulin (BAT). Representative images for all proteins are shown; all the bands for each picture always came from the same gel, although they may have been spliced for clarity, as represented by vertical lines.

### 4.8. In Situ Hybridization 

Coronal brain sections (16 µm) were probed with a specific oligonucleotide for prepro-OX (GenBank Accession Number: NM_013179; 5′-TTCGTAGAGACGGCAGGAACACGTCTTCTGGCGACA-3′) as previously published [12,44,45]. Sections were scanned, and the hybridization signal was quantified by densitometry using ImageJ-1.53 software (National Institutes of Health; Bethesda, MD, USA). We used 6–9 animals per experimental group and 16–24 sections for each animal (4–6 slides with four sections per slide). The mean of these 16–24 values was used as the densitometry value for each animal. 

### 4.9. Statistical Analysis

Data are presented as mean ± SEM. When two groups were compared, statistical significance was determined by a two-sided Student’s *t*-test; when more than groups were compared, statistical significance was determined by ANOVA followed by Bonferroni’s test. *p* < 0.05 was considered significant. Statistical analyses were performed using GraphPad Prism 8.0.2. Software (GraphPad Software Inc; San Diego, CA, USA).

## Data Availability

The data presented in this study are available on request from the corresponding author.

## Supplementary Materials

The following are available online at https://www.mdpi.com/1422-0067/22/4/1515/s1.

## Abbreviations

AMP	AMP-activated protein kinase
AKT	protein kinase B
BAT	brown adipose tissue
BMP8B	bone morphogenic protein 8B
δOR	ẟ opioid receptor
ERK1/2	p44/42 MAPK
ICV	intracerebroventricular
κOR	kappa opioid receptor
KO	knockout
LHA	lateral hypothalamic area
MAPK	mitogen-activated protein kinase
MCH	melanin-concentrating hormone
nAChR	nicotinic acetylcholine receptors
µOR	µ opioid receptor
norBNI	nor-Binaltorphimine dihydrochloride
OX	orexin
OXR1	orexin receptor 1
PKCζ	protein kinase C zeta
PVDF	polyvinylidene difluoride membranes
S6K	p70S6 kinase
SAPK/JNK	phospho-stress-activated protein kinase/c-Jun N-terminal kinase
SC	subcutaneous
SNS	sympathetic nerve system
rpS6	ribosomal protein S6
UCP1	uncoupling protein 1
VMH	ventromedial hypothalamic nucleus
WAT	white adipose tissue
