# Supplementary material for: κ-Opioid Signaling in the Lateral Hypothalamic Area Modulates Nicotine-Induced Negative Energy Balance
Source: Int J Mol Sci. 2021 Feb 3;22(4):1515. doi: 10.3390/ijms22041515 (PMC7913331; doi:10.3390/ijms22041515)
Supplement: Supplementary file 1 [file ijms-22-01515-s001.pdf]

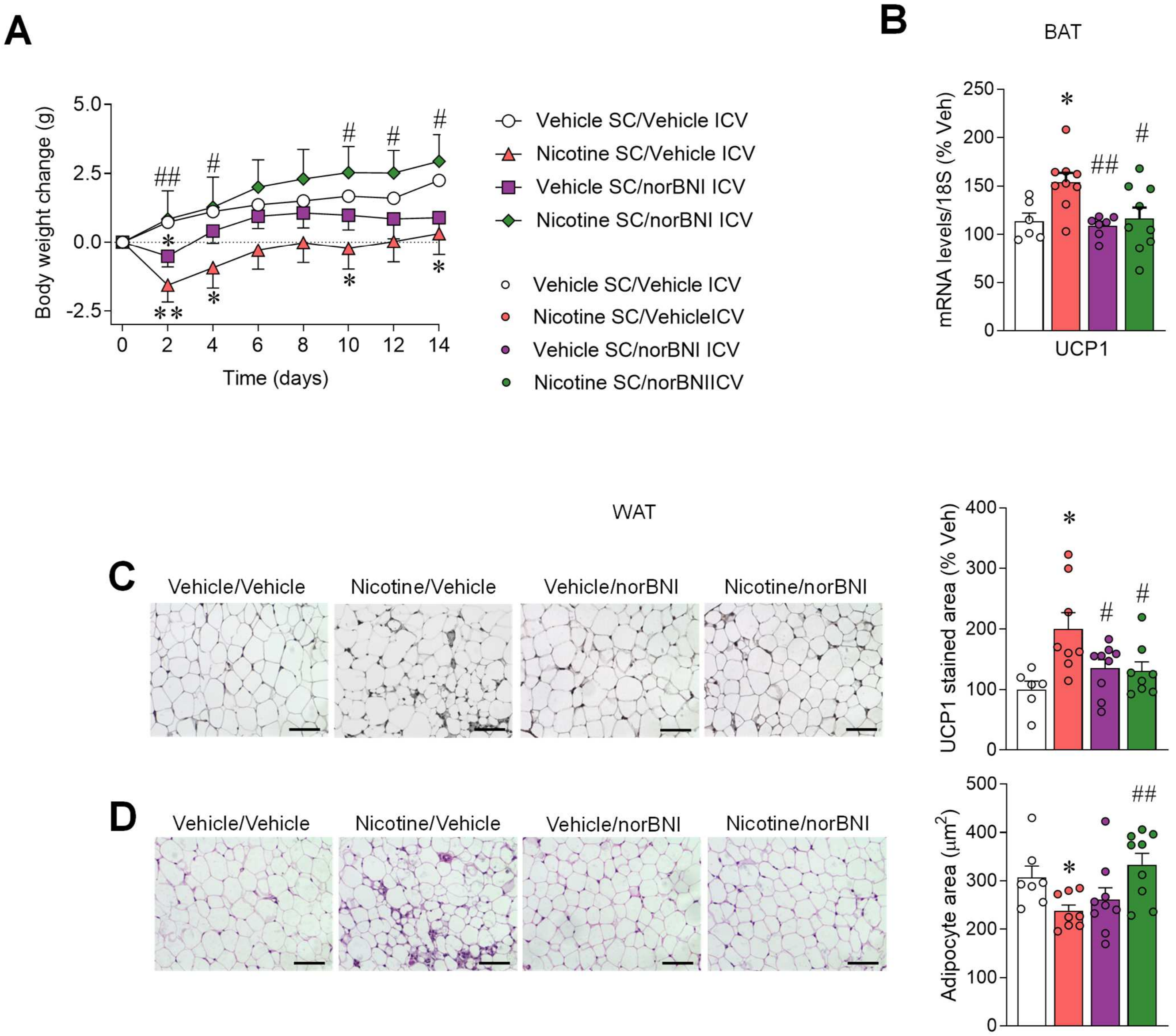

Supplemental Figure 1. Effect of pharmacological inhibition of κOR on nicotine-induced changes in energy balance
(**A**) Body weight change (n= 7-9 mice/group)
(**B**) mRNA levels of UCP1 in the BAT (n= 6-9 mice/group)
(**C**) UCP1 stained area in WAT (n= 6-9 mice/group)
(D) Adipocyte area in WAT (n= 7-9 mice/group)
of mice ICV treated with vehicle or norNBI and SC with vehicle or nicotine through osmotic minipumps for 14 days. *P<0.05, **P<0.01 *vs.* Vehicle SC/Vehicle ICV; # P<0.05, ## P<0.01 Nicotine SC/Vehicle ICV *vs.* Nicotine SC/norBNI ICV. Data expressed as mean ± SEM. scale bar: 100 μm.

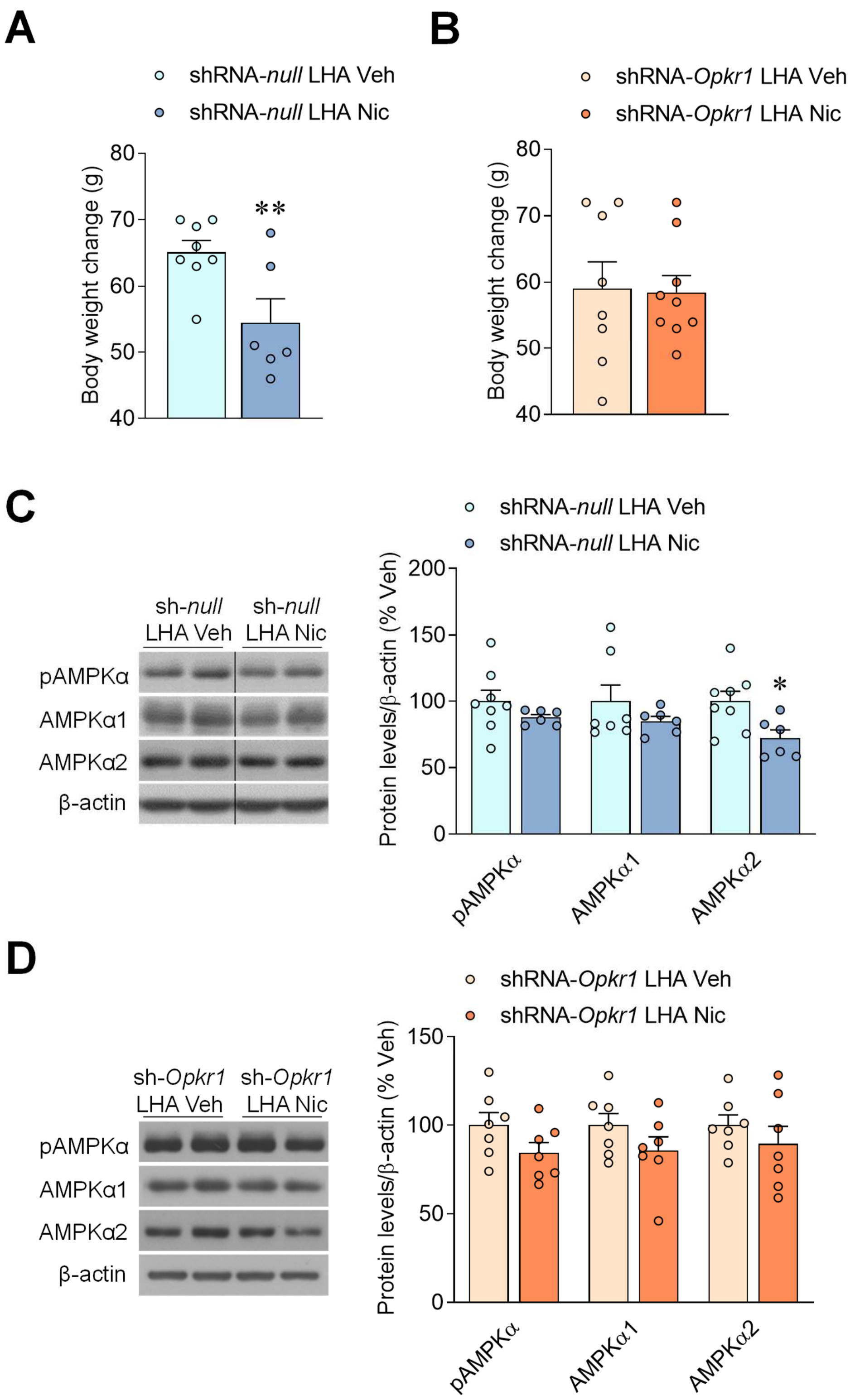

Supplemental Figure 2. Nicotine effect in energy balance is independent of AMPK pathway in the LHA
(A-B) Body weight change (n= 6-9 rats/group)
(**C-D**) Protein levels of the hypothalamic AMPK pathway (n= 6-8 mice/group)
of rats stereotaxically treated within the LHA with shRNA-*null* **(A and C)** or shRNA-*Opkr1* **(B and D)** adeno-associated viruses and treated SC with vehicle or nicotine through osmotic minipumps for 14 days.
